# Human papillomaviral load changes in low-grade squamous intraepithelial lesions of the uterine cervix

**DOI:** 10.1038/sj.bjc.6603430

**Published:** 2006-10-24

**Authors:** C-M Ho, W-F Cheng, T-Y Chu, C-A Chen, M-H Chuang, S-F Chang, C-Y Hsieh

**Affiliations:** 1 Gynecologic Cancer Center, Department of Obstetrics and Gynecology, Cathay General Hospital, Taipei, Taiwan, ROC; 2 School of Medicine, Fu Jen Catholic University, Hsinchuang, Taipei, Hsien, Taiwan, ROC; 3 Graduate Institute of Cell and Molecular Biology, Taipei Medical University, 250 Wu-Hsing Street, Taipei, Taiwan 110, ROC; 4 Department of Obstetrics and Gynecology, National Taiwan University Hospital, National Taiwan University, Taipei, Taiwan, ROC; 5 Department of Obstetrics and Gynecology, Buddhist Tzu Chi General Hospital, Hualien, Taiwan, ROC; 6 Division of Biostatistics and Bioinformatics, National Health Research Institutes, Miaoli, Taiwan, ROC

**Keywords:** HPV, low-grade squamous intraepithelial lesions, viral load, integration, hybrid capture 2

## Abstract

To better predict risk of progression of low-grade squamous intraepithelial lesions (LSILs) of the uterine cervix in women with human papillomavirus (HPV) infections, 294 baseline cervical specimens from women with LSILs were evaluated. Specimens were tested for HPV DNA using hybrid capture 2 (HC2) and PCR-reverse line blotting. 65 LSILs with HPV DNA types 16, 18, 52, or 58 were examined for physical status, E2/E6 ratio and viral load at two time points, along with patient age. Women with LSILs whose viral loads increased between baseline and 6 month follow-up had a 45% risk of developing HSIL (OR=7.6, 95% CI=1.9–29.4, *P*<0.01), as evaluated by real-time PCR and a 44% risk (OR=6.1, 95% CI=1.6–22.7, *P*<0.01), as evaluated by HC2. The two viral load measures correlated well (Person's coefficient, *r*=0.687, *P*<0.001). Such evaluations of viral load changes (increased or not increased) through repeat HPV DNA testing could predict progression of disease in LSIL cases of HPV types 16, 18, 52, and 58, which correlates to clinical implications.

Human papillomavirus (HPV) plays an important role in the carcinogenesis of cervical cancer (Bosch *et al*, 1995; Walboomers *et al*, 1999). The most common oncogenic HPV type in preinvasive and cervical cancer is HPV 16, detectable in 21% of women with low-grade squamous intraepithelial lesions (LSILs) (Castle *et al*, 2005), in more than 50% of women with cervical intraepithelial neoplasia grade 3 (CIN 3), and women with cervical cancer in America and Europe (Bosch *et al*, 1995; Herrero *et al*, 2000; Munoz *et al*, 2003). HPV 18 causes 10–15% of CIN 3 and cervical cancers (Bosch *et al*, 1995). In addition, HPV 18 also causes more than 35% of cervical adenocarcinomas, which are difficult to detect by current screening methods (Bosch *et al*, 1995). Additionally, HPV 52 and 58 are as prevalent as HPV 16 and 18 in patients with preinvasive and cervical cancer in Taiwan and Asian countries (Liaw *et al*, 1995; Huang *et al*, 1997; Ho *et al*, 2005, 2006).

Approximately 50% of atypical squamous cells of undetermined significance (ASCUS) and 80% of LSILs are infected by oncogenic types of HPV (ALTS group, 2000; Solomon *et al*, 2001). HPV DNA testing for patients with ASCUS provides useful information and allows referral of patients for immediate colposcopy to detect high-grade squamous intraepithelial lesions (HSILs) and cancer (Solomon *et al*, 2001). In contrast, oncogenic HPV DNA testing is not informative for triage of patients with LSILs because a high percentage of LSIL patients are HPV positive (ALTS group, 2000). A repeat Pap smear in 3–6 months or direct biopsy under colposcopy is generally used in clinical practice. Development of alternative triage strategies for women with LSILs would be valuable in distinguishing women with LSILs that have high probabilities of progression to HSILs from women with LSILs that have spontaneously regressed.

As a result of the lack of sufficiently large prospective longitudinal follow-up studies and the different geographic distributions of HPV types, it has not been established how the risk of HSILs differs during transition by physical status of HPV 16, 18, 52, and 58 DNA (integrated versus episomal DNA) during longitudinal follow-up, viral loads (high *vs* low) and viral load change (increased *vs* not increased) between baseline and follow-up among women with LSILs. To examine this issue, we evaluated the 2-year cumulative risk for HSIL attributable to HPV 16, 18, 52, and 58, the most common oncogenic types in pre-invasive cervical lesions including LSILs and HSILs in Asia, and questioned whether the integration of HPV oncogenes into a host genome contributed to the risk of LSILs progressing to HSILs. In addition, we determined if E6 viral load and its change contributed to the risk of LSILs progressing to HSILs during the 6-month interval between baseline diagnosis of LSIL by Pap smear and the 6-month follow-up visit by repeat Pap smear.

## SUBJECTS AND METHODS

The Taiwan Cooperative Oncology Group (TCOG), conducted a multicenter study – T1899, entitled, ‘Human Papillomavirus DNA in Squamous Intraepithelial Lesions and Longitudinal Follow-up of Its Clinical Significance in Low-grade Squamous Intraepithelial Lesions of Uterine Cervix’, under the supervision of the National Health Research Institute of Taiwan. A total of 1246 women with abnormal Pap smears, including those diagnosed with ASCUS or AGUS (*n*=431), LSIL (*n*=437), and HSIL (*n*=373), from August 1999 to March 2004 were enrolled. The details of the study design and population have been published (Sun *et al*, 2005; Chen *et al*, 2006). Of the 1246 participants, 936 underwent cervical biopsies for histological examinations.

Women with LSIL (*n*=437) had Pap smears, HPV testing, and colposcopic examinations every 3 months during the follow-up period. Participants were excluded from the study if they had no follow-up data (*n*=60), baseline cancer on pathological examination (*n*=1), fewer than four follow-up visits (*n*=82), or no baseline HPV data (*n*=2). Women with histologically confirmed HSIL were defined as having disease progression were treated and exited from the study. Women with LSIL with two consecutive normal Pap smears and showed HPV clearance during the follow-up period were defined as in remission and also exited. Women with LSIL not in remission or progression were defined as having persistent disease. HPV persistence was defined as HPV positivity for a given type tested on two consecutive occasions *vs* clearance.

A total of 294 women with LSIL, having at least four follow-up visits every 3 months, were tested for HPV DNA using both HC2 and PCR-reverse line blotting and were included in our longitudinal follow-up study (unpublished data). Among the 294 patients with LSIL, 187 specimens from 65 women with HPV 16 (*n*=14), 18 (*n*=8), 52 (*n*=30), and 58 (*n*=13) were collected at baseline and followed-up every 6 months until follow-up showed disease progression. Patients with LSIL from HPV types other than HPV 16, 18, 52, or 58 were excluded from our study due to diffuse distribution and relatively small sample sizes in each type. The specimens were further tested for viral load, E2/E6 ratio and viral load change using real-time PCR. Of the 187 specimens, 12 (6%) were mixed infections. Four samples lacking of follow-up HPV DNA were excluded from final analysis. In addition, 212 HSIL positive patients with HPV 16 (*n*=92), 18 (*n*=5), 52 (*n*=57), or 58 (*n*=58) infections were also obtained and tested for viral load, E2/E6 ratio, and physical status of HPV DNA using real-time PCR to compare with data from LSIL specimens. We also tested the specimens for viral load and viral load change by hybrid capture two and compared these results to those obtained by real-time PCR to predict disease progression.

### HPV DNA testing-HC2 and PCR methods

HC2 (Digene Corporation, Silver Spring, MD, USA) was used to examine for HPV DNA in each specimen, including a mixture of probes for cervical cancer-associated HPV types: 16, 18, 31, 33, 35, 39, 45, 51, 52, 56, 58, 59, and 68. The US Food and Drug Administration approved threshold of 1 pg of HPV DNA ml^−1^ of test solution was the positive control. The viral loads were expressed as copies of HPV genomes/100 ng. For the PCR method, MY09-MY-11-HMB01 L1 consensus primers were used in the amplification system, followed by a single hybridisation with a reverse line blot detection method (the prototype of Roche linear array HPV test, Roche Molecular Systems Inc., Pleasanton, CA, USA). The HPV genotyping strips contained 29 probe lines plus one reference line, detecting 27 individual HPV genotypes and two concentrations of the *β*-globin control probe. Two bovine serum albumin (BSA)-conjugated probes per HPV probe per HPV type, corresponding to each of two hypervariable regions within the MY09/MY11 amplicon, were deposited in a single line for each of the following HPV types: 16, 18, 26, 31, 33, 35, 39, 42, 45, 51–59, 66, 68, MM4, MM7, MM8, and MM9.

### Real-time PCR

DNA amplifications were carried out in a 96-well reaction plate format in an ABI Prism 5700 Sequence Detection System. Amplification and quantification of the E2 and E6 genes were performed simultaneously in separate reaction tubes. Both the HPV E2 and E6 PCR reactions were performed in triplicate. Multiple negative water blanks were included in every analysis. The reactions of real-time PCR are described elsewhere (Ho *et al*, 2006). The specificity was verified by a dissociation curve. Two standard curves were obtained by amplification of a serial dilution of cloned HPV-16 (from base 28 to base 3890) or HPV-18 (from base 45 to base 3993) DNA fragments or HPV-52 (from base 95 to base 3895) or HPV-58 (from base 45 to base 3994) DNA in pGEM T-Easy vector containing equivalent amounts of E2 and E6 genes from 10 to 10 000 000 copies *μ*l^−1^. The number of threshold cycles obtained from E2 PCR and from E6 PCR was equivalent in each run. Linear plots of the log of copy number *vs* the number of the threshold cycle were consistently obtained for both genes, and the correlation coefficient was between 0.995 and 1.00 in each run.

The technique was developed on the assumption that: (1) preferential disruption of E2 causes the absence of E2 gene sequences in the PCR product following integration, (2) copy numbers of both genes should be equal when viral DNA presents in episomal forms, and (3) E2 gene copy numbers are smaller than that for E6 in concomitant forms.

Concentrations of HPV DNA were expressed as copies of the HPV genome in 100 ng of cellular DNA. Ratios of E2–E6 of less than 1 indicated the presence of both integrated and episomal forms. The integrated E6 was calculated by subtracting the copy numbers of E2 (episomal). The ratio of E2 to integrated E6 represented the amount of the episomal form in relation to the integrated form.

### Statistical analyses

The 2-year cumulative risks stratified by the change of viral load at the first two time points (viral load increased or not increased) for histologically confirmed HSIL attributable to HPV 16, 18, 52, or 58 were analysed via the Kaplan-Meier method. Logistic regression analysis was used to calculate the odds ratios (OR) and their 95% confidence intervals (CIs) for HSIL associated with change of HPV load (increased *vs* not increased), high viral load (>10^5^ *vs* ⩽10^5^), age (⩾ 30 *vs* <30 years) and repeat Pap smear (abnormal *vs* normal). The correlation between HPV loads determined by real-time PCR and HC2 was estimated using Pearson's correlation. The differences in HPV integration status between LSIL and HISL groups were analysed using Fisher's exact test. All statistical analyses were performed using SAS 9.13.

## RESULTS

Among the 294 women with LSIL having at least four follow-up visits in 3 months, 76.9% were HPV DNA positive by PCR. The frequencies of the major, baseline HPV genotypes were: HPV 16 (4.8%), 18 (2.4%), 52 (10.9%), 58 (4.1%), 53 (8.5%), and 51 (6.8%). The prevalence of HPV 16, 18, 52, and 58 in HSIL were as follows: 31.7% (92/290) – type 16, followed by 20.0% (58/290) – type 58, 19.7% (57/290) – type 52, and 1.7% (5/290) – type 18.

### Median E6 viral loads

For the 61 eligible women with LSILs, the clinical characteristics and median viral loads are shown in Table 1. The median viral loads of HPV types 16 and 52 among women with LSILs and HSILs differed significantly (*P*=0.049 for HPV 16 E6, *P*=0.032 for HPV 52 E6). The median viral loads of HPV types 18 and 58 among women with LSILs and HSILs did not differ significantly (*P*=0.499 for type 18, *P*=0.184 for type 58). The median viral load ranged from 1.3 × 10^4^ to 3.7 × 10^4^ copies per 100 ng of DNA. Therefore, high viral loads were defined as those greater than 10^5^ copies per 100 ng of DNA. The HPV 16, 18, 52, or 58 viral loads tested by real-time PCR varied from 2.4 × 10^2^–7.6 × 10^6^ copies per 100 ng of DNA, which is equivalent to 15 151 cells. Whereas, the HPV 16, 18, 52, or 58 loads tested by HC2 varied from 5.1 × 10^3^–1.1 × 10^8^ copies per 100 ng of DNA, which is equivalent to about 15 000 cells. Women who had high viral loads (>10^5^ copies) *vs* low viral loads (⩽10^5^ copies) at baseline had marginally significant increased risk of disease progression (*P*=0.07).

### Two-year cumulative risk with dynamic change of HPV

**Figure 1:**
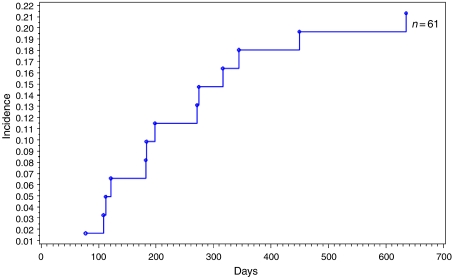
Kaplan–Meier estimates of cumulative incidence of high-grade squamous intraepithelial lesions (HSILs) among the 61 women with low-grade squamous intraepithelial lesions (LSILs), according to HPV types 16, 18, 52, and/or 58.

Overall, 61 women after cytological examination with LSILs attributable to HPV 16, 18, 52, or 58 had a 2-year cumulative risk of developing HSILs of 21.5%, 29.3% (4/14) with HPV 16 (OR=1.5, 95% CI=0.4–5.8, *P*=0.34), 12.5% (1/8) with HPV 18 (OR=0.6, 95% CI=0.1–5.3, *P*=0.23), 26.9% (7/26) with HPV 52 (OR=0.7, 95% CI=0.2–2.6, *P*=0.34), and 7.7% (1/13) with HPV 58 (OR=0.3, 95% CI=0.0–2.1, *P*=1.61) (Figure 1).

**Figure 2:**
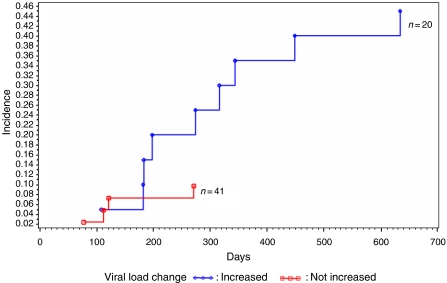
Kaplan–Meier estimates of cumulative incidence of high-grade squamous intraepithelial lesions (HSILs) among the 61 women with low-grade squamous intraepithelial lesions (LSILs), according to viral load change of HPV for HPV 16, 18, 52, and 58 (load increased *vs* not increased).

**Figure 3:**
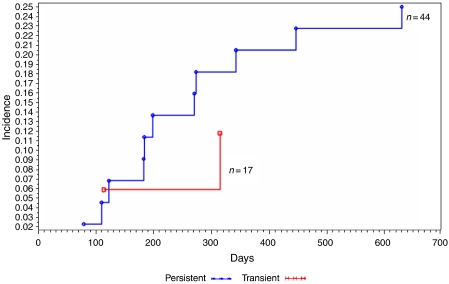
Kaplan–Meier estimates of cumulative incidence of high-grade squamous intraepithelial lesions (HSILs) among the 61 women with low-grade squamous intraepithelial lesions (LSILs), according to transient *vs* persistent infection of HPV with HPV 16, 18, 52, and/or 58.

Based on viral load changes detected using real-time PCR, women with LSILs who had viral loads increases for HPV 16, 18, 52, or 58 between baseline and a 6 month follow-up had a 2-year risk of developing HSIL of 45%, seven-fold greater than those without increases in viral loads (OR=7.6, 95% CI=1.9–24.3, *P*<0.01) (Figure 2). Overall, women with LSILs and persistent HPV infections had a 2-year cumulative risk of developing HSILs of 25%, compared –11.5% for those with transient infections (OR=2.5, 95% CI=0.5–12.7, *P*=0.27) (Figure 3).

Women with LSILs due to HPV 16, 18, 52, or 58, who had increases in viral loads between baseline and 6 month follow-up had risk of persistent abnormal Pap smears 45-fold greater than those without increased viral loads (OR=45.6, 95% CI=6.4–326.0, *P*<0.001).

Using logistic regression for modelling changes in viral load data, repeat abnormal Pap smears, and patient age at enrollment, women with LSILs who had viral loads increase between baseline and 6 month follow-up for HPV 16, 18, 52, or 58 had increased risk for HSILs, as tested by real-time PCR (OR=8.3, 95% CI=2.0–34, *P*<0.01) or HC2 (OR=5.6, 95% CI=1.5–21.4, *P*<0.01). In addition, women with LSILs, repeat abnormal Pap smears (OR=2.1, 95% CI=0.5–8.3, *P*>0.05), and aged younger than 30 years (OR=1.8, 95% CI=0.3–11.7, *P*>0.05) did not show an increase in risk of developing HSILs (Table 2).

### Comparison of HPV viral load by HC2 and real-time PCR

Real-time PCR and HC2 correlated well for determining viral load change (increased or not increased) at baseline and 6-month follow-up to predict disease progression. Based on viral load change tested by HC2, women with LSILs due to HPV 16, 18, 52, or 58 who had viral load increase between baseline and 6 month follow-up had risk of developing HSILs of 44%, six-fold greater than those without increased viral loads (OR=6.1, 95% CI=1.6–22.7). This result is consistent with real-time PCR of 45%, seven-fold greater than those without increased viral loads by real-time PCR. The logarithm of HPV 16, 18, 52, or 58 viral loads estimated by real-time PCR were plotted as functions of the logarithm of HPV viral loads estimated by HC2. The two measurements for viral load correlated well (Pearson's correlation coefficient *ρ*=0.69, *P*<0.001).

### Physical status of HPV 16, 18, 52, and 58 DNA in women with LSILs and longitudinal follow-up

The prevalence of both integrated and episomal (mixed type) infections for women with LSILs at baseline for HPV 16, 18, 52, and 58 were as follows: 7.1% (1/14) – type 16, 12.5% (1/8) – type 18, 7.7% (2/26) – type 52, and 0% (0/13) – type 58. No pure single type of viral integration was found in women with LSILs due to HPV 16, 18, 52, and 58 at baseline or during follow-up. Integrated and episomal HPV 16, 18, 52, or 58 DNA was detected in only 1 of 13 (7.7%) specimens in progression among women during transition from LSILs to HSILs. The prevalence of integration of HPV 16, 18, 52, or 58 DNA between women with LSILs and HSILs increased significantly (*P*<0.001). A significant difference or trend in the physical status of HPV 52, 18, or 58 DNA was found between patients with LSILs and HSILs (*P*<0.001 for type 52, *P*=0.067 for type 18, and *P*=0.065 for HPV 58). However, no significant difference in the physical status of HPV 16 DNA was found between patients with LSILs and HSILs (*P*=0.46). When the comparison was confined to tissue proved HSIL cases, the prevalence of integration of HPV 16, 18, 52, or 58 DNA between LSIL and HSIL cases was still significantly increased (*P*<0.001). A significant difference or trend in the physical status of HPV 52 or 58 DNA was found between patients with LSILs and HSILs (*P*<0.001 for type 52, and *P*=0.082 for HPV 58). However, the physical status of HPV 16 and 18 DNA was not significantly different between patients with LSILs and HSILs (*P*=0.3 for type 16 and *P*=0.22 for type 18).

## DISCUSSION

In our study population of women with LSILs, viral loads for HPV 16, 18, 52, and 58 increased during the 6 months of follow-up after baseline values were obtained. This increase in viral load was associated with a high risk of developing HSILs over a cumulative 2-year period, a seven-fold higher risk estimated by real-time PCR and a six-fold greater risk by HC2 compared to patients whose viral loads did not increase during the follow-up period.

We observed a 45%, 2-year cumulative risk of clinically relevant HSIL for HPV 16, 18, 52, and 58 in women with LSILs and whose viral loads increased between baseline and 6-month follow-up. Moreover, women with LSILs who had viral load increase between baseline and 6 month follow-up for HPV 16, 18, 52, or 58, had risk of persistent abnormal Pap smear 45-fold greater than did those who had no increase in viral loads (OR=45.6, 95% CI=6.4–326.0, *P*<0.001). The frequent monitoring of these women allowed an early diagnosis of high-risk (HR) HPV, and thereby, early therapeutic intervention. Indeed HR HPV and especially HPV 16, 18, 52, and 58 infections persisted and progressed throughout the follow-up period. Van Duin *et al* (2002) reported similar results and hypothesised that increasing viral loads over time would favour viral persistence, and the development of cervical CIN 2/3 or worse.

Women with LSIL cytology who were HPV 16 DNA positive at baseline did not have significantly increased risk of developing HSIL (OR=1.5, 95% CI=0.4–5.8). One possible explanation for this is the number of women with LSILs due to HPV 16 was limited (only 9%), compared to 21% in Castle *et al* (2005). We observed that high viral loads (>10^5^ copies) at baseline reached marginal significance (*P*=0.07) in predicting disease progression among women with LSILs, consistent with Castle *et al* (2005). Additionally, neither repeat abnormal Pap smear nor age older than 30 years increased the risk of HSIL. Thus, increased HPV 16, 18, 52, and 58 viral loads was the single most important risk factor for HSIL in our study population.

The increase of viral load detected by both HC2 and real-time PCR methods predicted disease progression to HSIL, with HPV 16, 18, 52, and 58 viral loads detection correlating well (only 6% of tested specimens had mixed HPV types) and similar to Prétet *et al* (2004). We used a real-time PCR method for E6 HPV 16, 18, 52, and 58 quantifications to determine whether the HC2 assay could be suitable as a quantitative viral load assay for clinical practice. Recent data suggests that HR-HPV load would help to identify women at high risk for progression to HSIL (Joseffson *et al*, 2000; Lorincz *et al*, 2002). However, due to multiple infections, cross-reactivity of nononcogenic HPV types, and variability of cellularity of cervical scrapings, HC2 assay of HR-HPV load has not been validated as a quantitative test.

Although, specimens with HPV 16, 18, 52, and 58 represent about 43% of all LSILs (Chen *et al*, 2006), they represented 73% of HSILs in our cohort study. As a result of the relatively small proportion of HPV 16 in women with LSILs in Taiwan, it seems that distinguishing the risk of cervical precancer among HPV 16-positive women from the lower risk posed by other oncogenic HPV types might have limited clinical value. Alternatively, repeat HPV DNA testing 6 months after baseline testing to determine changes in viral load (increased or not increased) could predict progression of disease among women with LSILs due to HPV types 16, 18, 52, and 58, and might be clinically practical and valuable. Although, it is useful for ASCUS triage (Solomon *et al*, 2001), HC2 is not recommended for LSIL triage because the high proportion of positive results makes it uninformative (ALTS group, 2000). Nonetheless, if the elevated risk of HSILs in patients who are positive for HPV 16, 18, 52, or 58 and have viral load increases over the first two time points at 6-month interval (33% of patients who are positive for HPV 16, 18, 52, or 58) warrants more aggressive treatment, then determination of viral load change (increase) of individual HPV 16, 18, 52, and 58 types might be useful for the management of women with LSILs. It is noteworthy that women who had undetectable HPV or viral loads at 1 log decreased between baseline and 6 months of follow-up still had 6% (2/34) risk of developing HSILs (data not shown). These data highlight that no single test or combination of tests will provide perfect negative reassurance of cervical precancer or cancer.

Several authors also note that the quantitative measurement of HPV DNA, as an evolutional predictor or determinant of disease severity, is of greater value than a single HPV positive result. For example, the amount of HR HPV DNA increases the grade of the lesion. This increase was noted for HPV 16, but not for HPV 18, 31, 33, 45, and 56 (Moberg *et al*, 2004). Moreover, the highest HPV 16 loads are predictive of the appearance of carcinoma *in situ* before any intraepithelial lesions are detectable (Joseffson *et al*, 2000). In a model of the temporal relationship in HPV-induced carcinoma, Peitsaro *et al* (2002) proposed that the probability of HPV DNA integration increases with high HPV 16 DNA load. In our study, the median viral loads of HPV type 16 and 52 in women with cytologies between LSIL and HSIL differed significantly, in contrast to those of HPV types 18 and 58. In our study, we could not compare the viral loads between women with integration in LSILs and women with episomal in LSILs, because of the low integration rate in LSILs cases. However, we compared the median viral loads of E6 between women with integration in primary HSILs and women with episomal in primary HSILs. There were no significant differences in pooling data (including HPV 16, 18, 52, or 58) or different type between these two groups of patients (data not shown).

The value of 1 pg of HPV genome ml^−1^, which represents the HC2 assay threshold, corresponds to 10^5^ copies of HPV genome ml^−1^. As the cervical samples harboured a mean of 5 × 10^5^ cells ml^−1^, the HC2 cutoff represents 10^5^ HPV/5 × 10^5^ cells. Van Duin *et al* (2002) proposed that it was 2.4 × 10^4^ HPV 16/scrape (one scrape contains approximated 0.6 × 10^5^–2.2 × 10^5^ cells) for women with normal cytologies and 4.3 × 10^6^ HPV 16/scrape for women with abnormal cytologies. The real-time PCR and HC2 measurements of viral load correlated well, even without cell normalization in our study (Pearson's correlation coefficient *r*=0.69, *P*<0.001). Load changes determined by HC2 also precisely predicted histological progression of LSILs to HSILs.

In order to elucidate the causal relationship of integration in LSIL progression to HSILs using real-time PCR method, we analysed HPV 16, 18, 52, and 58 DNA samples in 178 cervical swabs from women with LSILs and longitudinally monitored them every 3 months in a multicentre trial. We detected the integrated form only in 9/178 (5%) samples with the episomal form. Additionally, no pure integration was found in women with LSILs due to HPV 16, 18, 52, and 58 at baseline and during follow-up. Furthermore, integration (both integrated DNA and episomal DNA) of HPV 16, 18, 52, or 58 DNA was detected in only 1 of 13 (7.7%) specimens of women during transition from LSILs to HSILs. Although some studies (Kulmala *et al*, 2006) reported that early integration is noted in women with LSILs, our data suggests that integration is not often detected in women with LSILs, and probably is not essential for progression during transition from LSILs to HSILs. On the other hand, the prevalence of integration of HPV 16, 18, 52, or 58 DNA among women progressing from LSILs to HSILs is significantly increased (*P*<0.001), which is comparable with previous studies showing that integration is often detected in HSILs (Peitsaro *et al*, 2002). Although, there was no difference for HPV 16 infection between women with LSILs and HSILs, this may be due to small sample size or reasons not yet known. The possible role of HPV DNA integration may be a late event in carcinogenesis, especially when it occurs in HSILs or cervical cancer, or when LSIL skips directly to HSIL after persistent HPV infection.

In conclusion, we recommend that patients with LSIL, HPV types 16, 18, 52, or 58, consider detecting the viral load with real-time PCR or HC2 at the first visit and 6 months subsequently. Those with elevated viral loads are at risk of disease progression and should be carefully evaluated by colposcopy.

## Figures and Tables

**Table 1:** Clinical characteristics and median E6 load for LSIL of 61 eligible women with LSILs due to HPV 16, 18, 52, or 58 with at least four follow-up visits

	**HPV 16**	**HPV 18**	**HPV 52**	**HPV 58**
Percentage of each type	23.0% (14/61)	13.1% (8/61)	42.6% (26/61)	21.3% (13/61)
Median age (range)	35 (range: 24–58)	31 (range: 23–51)	41 (range: 24–65)	38 (range: 20–52)
Median E6 load for LSIL	13 437	26 599	35 620	37 366
Range of viral load for LSIL	0–735 527	0–5 029 143	0–3 843 486	0–2 754 113
Median E6 load for HSIL	31 916	21 067	156 906	110 107
Range of viral load for HSIL	0–34 386 322	0–41 748	0–13 596 437	0–14 243 132
*P*-value (median E6 load between LSIL and HSIL)	0.049	0.499	0.032	0.184

HSILs=high-grade squamous intraepithelial lesions.

**Table 2:** OR and 95% confidence intervals (95% CI) for 2-year cumulative HSIL diagnoses associated with HPV 16, 18, 52, and 58 status, viral load change, age, high viral load, and repeat Pap smear

**Characteristic**	**OR (95**% **CI)**	***P*-value**
*HPV risk*		
HPV 16 (*n*=14)	1.5 (0.4–5.8)	0.34
HPV 18 (*n*=8)	0.6 (0.1–5.3)	0.23
HPV 52 (*n*=26)	0.7 (0.2–2.6)	0.34
HPV 58 (*n*=13)	0.3 (0.0–2.1)	1.61
		
*Baseline viral load*		
HPV DNA>10^5^/or HPV DNA⩽l 10^5^	0.8 (0.2–3.1)	0.07
		
*Viral load change*		
*Viral load increase between baseline and 6-month follow-up for HPV testing*		
Real-time PCR	8.3 (2.0–34)	<0.01
Hybrid capture 2	5.6 (1.5–21.4)	<0.01
		
*HPV persistence*		
Persistent HPV/transient	2.5 (0.5–12.7)	0.27
		
*Age at enrollment*		
⩾30 or <30	1.8 (0.3–11.7)	0.19
		
*Repeat Pap smear*		
Abnormal or normal	2.1 (0.5–8.3)	1.27

*P*-value <0.05 represents statistical significance (*χ*^2^).
